# Noninvasive positive pressure ventilation after extubation: features and outcomes in clinical practice

**DOI:** 10.5935/0103-507X.20150046

**Published:** 2015

**Authors:** Liria Yuri Yamauchi, Maise Figueiroa, Leda Tomiko Yamada da Silveira, Teresa Cristina Francischetto Travaglia, Sidnei Bernardes, Carolina Fu

**Affiliations:** 1 Department of Human Movement Sciences, Universidade Federal de São Paulo - Santos (SP), Brazil.; 2 Department of Physiotherapy, Communication Sciences & Disorders, and Occupational Therapy, Faculdade de Medicina, Universidade de São Paulo - São Paulo (SP), Brazil.

**Keywords:** Respiration, artificial, Positive-pressure respiration/methods, Airway extubation, Ventilator weaning, Treatment outcomes, Intensive care units

## Abstract

**Objective:**

To describe postextubation noninvasive positive pressure ventilation use in intensive care unit clinical practice and to identify factors associated with noninvasive positive pressure ventilation failure.

**Methods:**

This prospective cohort study included patients aged ≥ 18 years consecutively admitted to the intensive care unit who required noninvasive positive pressure ventilation within 48 hours of extubation. The primary outcome was noninvasive positive pressure ventilation failure.

**Results:**

We included 174 patients in the study. The overall noninvasive positive pressure ventilation use rate was 15%. Among the patients who used noninvasive positive pressure ventilation, 44% used it after extubation. The failure rate of noninvasive positive pressure ventilation was 34%. The overall mean ± SD age was 56 ± 18 years, and 55% of participants were male. Demographics; baseline pH, PaCO2 and HCO3; and type of equipment used were similar between groups. All of the noninvasive positive pressure ventilation final parameters were higher in the noninvasive positive pressure ventilation failure group [inspiratory positive airway pressure: 15.0 versus 13.7cmH2O (p = 0.015), expiratory positive airway pressure: 10.0 versus 8.9cmH2O (p = 0.027), and FiO2: 41 versus 33% (p = 0.014)]. The mean intensive care unit length of stay was longer (24 versus 13 days), p < 0.001, and the intensive care unit mortality rate was higher (55 versus 10%), p < 0.001 in the noninvasive positive pressure ventilation failure group. After fitting, the logistic regression model allowed us to state that patients with inspiratory positive airway pressure ≥ 13.5cmH2O on the last day of noninvasive positive pressure ventilation support are three times more likely to experience noninvasive positive pressure ventilation failure compared with individuals with inspiratory positive airway pressure < 13.5 (OR = 3.02, 95%CI = 1.01 - 10.52, p value = 0.040).

**Conclusion:**

The noninvasive positive pressure ventilation failure group had a longer intensive care unit length of stay and a higher mortality rate. Logistic regression analysis identified that patients with inspiratory positive airway pressure ≥ 13.5cmH_2_O on the last day of noninvasive positive pressure ventilation support are three times more likely to experience noninvasive positive pressure ventilation failure.

## Abstract (pt)

**Objetivo:**

Descrever o uso de ventilação não invasiva com pressão positiva pós-extubação na prática clínica da unidade de terapia intensiva, e identificar os fatores associados à falência da ventilação não invasiva com pressão positiva.

**Métodos:**

Este estudo prospectivo de coorte incluiu pacientes com idade ≥ 18 anos admitidos consecutivamente à unidade de terapia intensiva e submetidos à ventilação não invasiva com pressão positiva dentro de 48 horas após sua extubação. O desfecho primário foi falência da ventilação não invasiva com pressão positiva.

**Resultados:**

Incluímos um total de 174 pacientes. A taxa global de uso de ventilação não invasiva com pressão positiva foi de 15%. Dentre todos os pacientes que utilizaram ventilação não invasiva com pressão positiva, em 44% o uso ocorreu pós-extubação. A taxa de falência da ventilação não invasiva com pressão positiva foi de 34%. A média de idade (± DP) foi de 56 ± 18 anos, sendo que 55% dos pacientes eram do sexo masculino. Os dados demográficos, níveis basais de pH, PaCO_2_ e HCO_3_ além do tipo de equipamento utilizado foram similares entre os grupos. Todos os parâmetros finais de ventilação não invasiva com pressão positiva foram mais elevados no grupo que apresentou falência da ventilação não invasiva com pressão positiva (pressão inspiratória positiva nas vias aéreas - 15,0 versus 13,7cmH_2_O; p = 0,015; pressão expiratória positiva nas vias aéreas - 10,0 *versus* 8,9cmH_2_O; p = 0,027; e FiO_2_ - 41 *versus* 33%; p = 0,014). O grupo que teve falência da ventilação não invasiva com pressão positiva teve tempo médio de permanência na unidade de terapia intensiva maior (24 versus 13 dias; p < 0,001), e taxa de mortalidade na unidade de terapia intensiva mais elevada (55 *versus* 10%; p < 0,001). Após adequação, o modelo de regressão logística permitiu afirmar que pacientes com pressão inspiratória positiva nas vias aéreas ≥ 13,5cmH_2_O no último dia de suporte com ventilação não invasiva com pressão positiva tiveram risco três vezes maior de se tornarem casos de falência da ventilação não invasiva com pressão positiva, do que os pacientes que tiveram pressão inspiratória positiva das vias aéreas < 13,5 (OR = 3,02; IC95% = 1,01 - 10,52; p = 0,040).

**Conclusão:**

O grupo com falência da ventilação não invasiva com pressão positiva teve tempo de permanência na unidade de terapia intensiva maior, além de uma taxa de mortalidade mais elevada. A análise de regressão logística identificou que pacientes com pressão inspiratória positiva nas vias aéreas ≥ 13,5cmH2O no último dia de suporte ventilatório não invasivo tiveram risco três vezes maior de apresentar falência da ventilação não invasiva com pressão positiva.

## INTRODUCTION

Noninvasive positive pressure ventilation (NIPPV) has been widely used in intensive care units (ICU). Despite conflicting scientific evidence regarding many indications for its use, NIPPV has become a part of routine care in the majority of ICU worldwide.^(1-4)^ According to the literature, some indications are considered acceptable, but others are still under investigation, such as the use of NIPPV after extubation.

This approach has some different nuances, mainly based on timing. Some studies have incorporated NIPPV into the weaning from invasive mechanical ventilation, meaning that NIPPV is applied immediately after extubation as part of a continuous process.^(5-8)^ In these cases, NIPPV can be applied immediately as a preventive,^(9,10)^ after failure of a spontaneous breathing trial^(11)^ or after extubation of high-risk patients.^(12)^

In other hand, the use of NIPPV after the development of acute respiratory failure (ARF) after extubation has presented conflicting results. While some studies have found that NIPPV may prevent reintubation,^(13)^ others have shown that it does not seem to diminish the reintubation rate and may even increase the mortality rate.^(14)^

From a clinical point of view, NIPPV is indispensable in the ICU, and information about its use in practice may raise some important issues not identified in randomized clinical trials. The present study was undertaken to describe post-extubation NIPPV use in ICU clinical practice and to identify factors associated with NIPPV failure after extubation.

## METHODS

Between May and December 2007, a prospective cohort study was conducted at *Hospital das Clínicas *of the* Faculdade de Medicina *of the* Universidade de São Paulo*, located in the city of São Paulo, Brazil. The study was carried out in eleven ICU (140 beds). This study was approved by the hospital Ethical Committee (number 0327/07), and the requirement for informed consent was waived because data were collected from patients’ records, and no intervention was performed.

All adult patients (age ≥ 18 years) consecutively admitted to the ICU who used NIPPV within 48 hours of extubation were included. Patients were excluded if there was any relevant information missing from the charts.

Data were collected from medical charts and directly from the ICU staff. All decisions about NIPPV use were exclusively made by the ICU team; researchers did not intervene in any way. Patients were analyzed as success NIPPV group and failure NIPPV group. The following data were collected: demographics [age, gender and Simplified Acute Physiology Score (SAPS II) at ICU admission]; day and time of intubation; reason for invasive mechanical ventilation [chronic obstructive pulmonary disease (COPD), asthma, decreased level of consciousness, neuromuscular disease, ARF, cardiac arrest, hemodynamic instability or surgery]; day and time of extubation; and day and time of the start of NIPPV.

Data related to NIPPV collected in the study included the indication for NIPPV [acute respiratory failure after extubation (signs of respiratory distress up to 48 hours after extubation), early weaning (NIPPV immediately after extubation in patients considered at high risk for reintubation, such as COPD patients), and preventive NIPPV (in cases without ARF but with relevant comorbidities)]; the period of NIPPV use; type of equipment used (BIPAP Vision - Respironics^®^, BIPAP ST/d Respironics^®^, Downs flow generator - Vital Signs^®^, or double function mechanical ventilator); time from extubation until NIPPV initiation (0 or ≥ 1 day); NIPPV parameters; type of NIPPV interface; arterial blood gas test prior to NIPPV use; mask leakage; intolerance to NIPPV; need for airway suctioning; NIPPV complications; reintubation rate; reasons for reintubation; NIPPV failure rate (defined as reintubation after NIPPV use); ICU mortality rate; and ICU length of stay.

### Statistical analysis

A descriptive analysis was carried out. Quantitative variables were presented as the mean and standard deviation (SD) or the median and interquartile range (IQR). Categorical variables were presented as proportions. The predictive capacity of quantitative variables for NIPPV failure was assessed with receiver-operating characteristic (ROC) curves; the area under the curve (AUC) and optimal cutoff values (based on best values of sensitivity and specificity) were calculated.

The logistic regression model was fitted using NIPPV failure as a dependent variable. The following steps were taken: independent variables were selected based on their clinical relevance and were dichotomized based on cutoff values calculated by ROC curves. After that, all independent variables were submitted to univariate analysis. Odds ratio and Fisher’s exact test were applied to identify possible associations among independent variables and NIPPV failure. The odds ratio of each independent variable was calculated based on 2 x 2 tables to define which variables would comprise the initial model of logistic regression. The variables with p-values above 0.30 were not included in the initial model. Multi-collinearity was evaluated by variance inflation factors. The Hosmer-Lemeshow test was applied to verify the goodness of fit model.

## RESULTS

**Figure 1:**
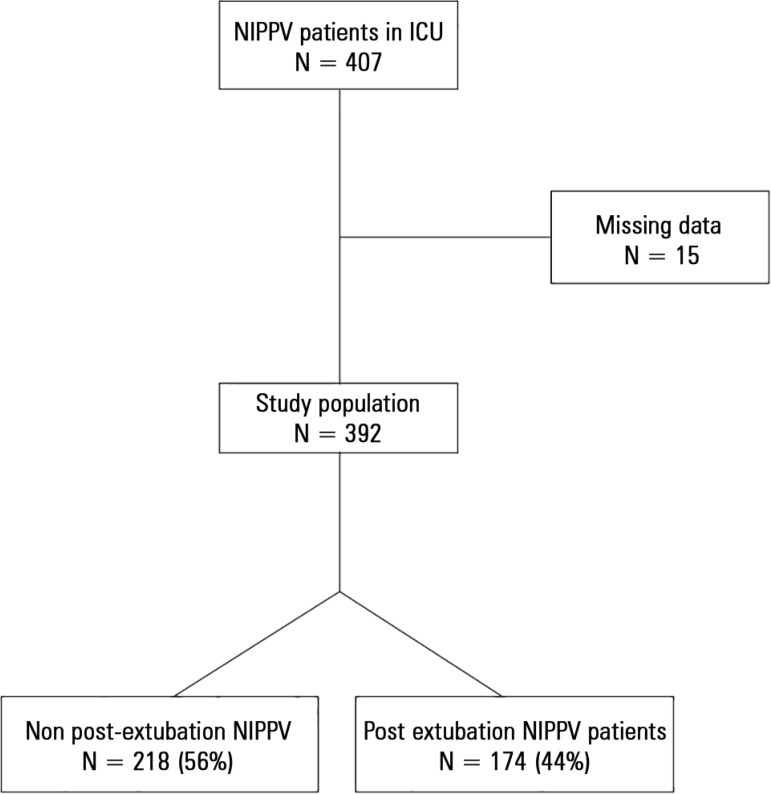
Study flowchart. NIPPV - noninvasive positive pressure ventilation; ICU - intensive care unit.

During the study period, 2,773 patients were admitted to the ICU. NIPPV was used on 407 (15%) of them. After excluding 15 patients due to missing data, the study population was 392 patients. Those who used NIPPV only after extubation accounted for 44%, or 174 patients (Figure 1). Baseline characteristics of the study population are presented in table 1.

**Table 1:** Baseline characteristics in patients treated with noninvasive positive pressure ventilation in the intensive care unit according to noninvasive positive pressure ventilation outcome

**Variables**	**All NIPPV patients N = 174**	**NIPPV success patients N = 114**	**NIPPV failure patients N = 60**
Age (years)	56 ± 18	55 ± 18	60 ± 17
Male	98 (56)	63 (55)	35 (58)
SAPS II at ICU admission	42 ± 18	40 ± 14	44 ± 14
Reason for ICU admission			
Medical	82 (47)	56 (50)	26 (42)
Emergency surgery	44 (25)	30 (26)	14 (23)
Elective surgery	48 (28)	28 (25)	20 (33)
Reason for initiation of mechanical ventilation			
Postoperative respiratory failure	56 (32)	36 (32)	20 (33)
Acute respiratory failure	42 (24)	27 (24)	15 (25)
ALI/ARDS	3 (2)	1 (1)	2 (3)
Cardiogenic pulmonary edema	4 (2)	2 (2)	2 (3)
Pneumonia	6 (3)	6 (5)	0 (0)
Trauma	15 (9)	10 (9)	5 (8)
Upper airway obstruction/Apnea	1 (0.6)	0 (0)	1 (2)
Other causes	12 (7)	8 (7)	4 (7)
Ignored	1 (0.6)		
Decreased level of consciousness	23 (13)	16 (14)	7 (12)
COPD	8 (5)	4 (3.5)	4 (7)
Cardiorespiratory arrest	3 (2)	3 (3)	0 (0)
Acute-on-chronic respiratory failure	2 (1)	2 (2)	0 (0)
Neuromuscular disease	1 (0.6)	0 (0)	1 (2)
Other	31 (18)	19 (17)	12 (20)
Ignored	2 (1)	1 (1)	1 (2)
Missing data	6 (3)		
pH at baseline	7.38 ± 0.1	7.38 ± 0.1	7.38 ± 0.05
PaCO_2_ at baseline (mmHg)	38.9 ± 8.9	39.8 ± 9.9	37.3 ± 6.3
HCO_3_ at baseline (mEq/L)	22.9 ± 5	22.8 ± 5.3	22.8 ± 4.7

NIPPV - noninvasive positive pressure ventilation; SAPS - Simplified Acute Physiology Score; ICU - intensive care unit; ALI - acute lung injury; ARDS - acute respiratory distress syndrome; COPD - chronic obstructive pulmonary disease; PaCO_2_ - partial pressure of carbon dioxide; HCO_3_ - bicarbonate. 7-test and chi-square test used as appropriate. The results are expressed in number (percentages) and mean ± standard deviation.

The main reasons for the use of mechanical ventilation prior to the use of NIPPV were hemodynamic instability (33%), acute respiratory failure (24%) and surgery (18%). The median (IQR) time of use of invasive mechanical ventilation was 4 (1 - 8) days. Noninvasive pressure ventilation features are presented in table 2. BIPAP Vision^®^ and continuous positive airway pressure flow generators were the most commonly used equipment. The main interface was the orofacial mask.

**Table 2:** Noninvasive positive pressure ventilation features according to noninvasive positive pressure ventilation outcome

**Variables**	**All NIPPV patients N = 174**	**NIPPV success N = 114**	**NIPPV Failure N = 60**
Type of equipment			
BIPAP Vision	75 (43)	43 (38)	32 (53)
BIPAP ST-D 30	11 (6)	8 (7)	3 (5)
CPAP flow generator	74 (42)	53 (46)	21 (35)
ICU ventilator	12 (7)	7 (6)	5 (8)
Other	2 (1)	2 (2)	0
Type of interface			
Oronasal mask	162 (93)	104 (91)	58 (97)
Facial	11 (6)	9 (8)	2 (3)
Nasal	1 (0.6)	1 (0.9)	0 (0)
Duration of NIPPV (hours)	34 (17 - 68)	30 (16 - 55)	50 (22 - 76)
NIPPV parameters in the last day			
CPAP (mmHg)	9.6 ± 1.2	9.6 ± 1.1	9.7 ± 1.6
IPAP (mmHg)	14.2 ± 2.3	13.7 ± 2.1	15 ± 2.3
EPAP (mmHg)	9.3 ± 2.1	8.9 ± 1.8	10 ± 2.4
FiO_2_ (%)	36 ± 12	33 ± 9.8	41 ± 15

NIPPV - noninvasive positive pressure ventilation; CPAP - continuous positive airway pressure; ICU- intensive care unit; IPAP - inspiratory positive airway pressure; EPAP - expiratory positive airway pressure; FiO_2_ - fraction of inspired oxygen. The results are expressed in number (percentages) and mean ± standard deviation.

NIPPV after extubation was applied in three situations: a new acute respiratory event [46 cases (26%)], early weaning [17 cases (10%)], and preventive NIPPV application [111 cases (64%)]. The time from extubation to initiation of NIPPV was recorded in days. A total of 121 patients (69%) received NIPPV support on the same day as extubation, and 53 (31%) received NIPPV between one and two days later.

During NIPPV support, the equipment was changed in some cases. At the beginning of NIPPV, the most commonly used device was a continuous positive airway pressure flow generator (45%) followed by BIPAP Vision^®^ (32%). However, on the last day of NIPPV, BIPAP Vision^®^ was more frequently used (42%).

All of the noninvasive positive pressure ventilation final parameters were higher in the noninvasive positive pressure ventilation failure group [inspiratory positive airway pressure: 15.0 versus 13.7cmH_2_O (p = 0.015), expiratory positive airway pressure: 10.0 versus 8.9cmH_2_O (p = 0.027), and FiO_2_: 41 versus 33% (p = 0.014)]. The mean intensive care unit length of stay was longer (24 versus 13 days), p < 0.001, and the intensive care unit mortality rate was higher (55 versus 10%), p < 0.001 in the noninvasive positive pressure ventilation failure group.

During the period of NIPPV use, 18% of patients presented with intolerance or excessive flow leakage, and treatment impairment occurred in 4%. NIPPV-related complications occurred in seven patients (five with vomiting, one with abdominal distention and one with skin lesions). The nosocomial pneumonia rate was 6%.

The NIPPV failure rate was 34%. The median time between extubation and reintubation was 2 (1 - 4) days. The main reasons for reintubation (NIPPV failure) were acute respiratory failure (48%) and decreased level of consciousness (22%). NIPPV failure did not differ according to indication: 32% were in the ARF after extubation group, 29% in the early NIPPV group and 35% in the preventive NIPPV group.

Patients with NIPPV failure presented a higher rate of tracheostomy [14 (23%) versus 0 (0%) patients, p < 0.001], a higher ICU length of stay [24 ± 15 versus 13 ± 7 days, p < 0.001], and a higher ICU mortality rate [33 (55%) versus 11 (10%), p < 0.001].

Independent variables were selected based on their clinical relevance, and continuous variables were dichotomized based on cutoff values calculated by ROC curves. The predictive power of all variables was not high. Area under the ROC curves, sensitivity and specificity calculated values are presented in table 3.

**Table 3:** Receiver operating characteristics curves results

**Variables**	**Cutoff values**	**Sensitivity (%) ** **(95%CI)**	**Specificity (%) ** **(95%CI)**	**AUC (95%CI)**
Age	59.5	63 (52; 75)	54 (44; 63)	0.56 (0.47; 0.65)
SAPS II score	36.5	70 (58; 80)	45 (36; 53)	0.59 (0.50; 0.68)
EPAP	9.5	71 (55; 84)	50 (36; 64)	0.64 (0.53; 0.76)
IPAP	13.5	81 (64; 93)	42 (28; 54)	0.64 (0.52; 0.76)
FiO_2_ (%)	37.5	57 (40; 73)	68 (54; 82)	0.65 (0.53; 0.78)

AUC - area under the receiver operating characteristic curves; 95%CI - 95% confidence interval; SAPS - Simplified Acute Physiology Score; EPAP - expiratory positive airway pressure; IPAP - inspiratory positive airway pressure; FiO_2_ - fraction of inspired oxygen.

Possible associations between the explanatory variables and dependent variables were also investigated. For this reason, the odds ratio of each variable was calculated, as presented in table 4. The multi-collinearity was investigated, and all the variance inflation factors were smaller than 2.

**Table 4:** Univariate analysis performed prior to the logistic regression

**Variable**	**OR (95% CI)**	p value*
Sex	0.79 (0.39 - 1.57)	0.522
Nasotracheal aspiration (yes or no)	1.69 (0.85 - 3.36)	0.108
Age ≥ 60	2.02 (1.01 - 4.06)	0.037
SAPS II > 36.5	1.88 (0.93 - 3.91)	0.073
Time to NIPPV start (days, 0 versus ≥1)	0.67 (0.30 - 1.41)	0.300
IPAP ≥ 13.5cmH2O	2.98 (0.96 - 10.48)	0.051
EPAP ≥ 9.5cmH2O	2.42 (0.86 - 7.21)	0.069
FiO_2_ ≥ 0.37	2.76 (0.96 - 8.18)	0.054

SAPS - Simplified Acute Physiology Score; NIPPV - noninvasive positive pressure ventilation; IPAP - inspiratory positive airway pressure; EPAP - expiratory positive airway pressure; FiO_2_ - fraction of inspired oxygen.

* Fisher’s exact test.

The variables selected to comprise the initial logistic regression model were need for nasotracheal suctioning (yes or no), age (< 60 or ≥ 60 years old), SAPS II score (< 36.5 or ≥ 36.5), expiratory positive airway pressure (EPAP) level on the last day of NIPPV (< 9.5 or ≥ 9.5cmH_2_O), inspiratory positive airway pressure (IPAP) level on the last day of NIPPV (IPAP < 13.5 or ≥ 13.5cmH_2_O), fraction of inspired oxygen (FiO_2_) level on the last day of NIPPV (FiO_2_ < 0.37 or ≥ 0.37), and time from extubation to NIPPV start (on the same day or ≥ 1 day). The Hosmer-Lemeshow test found a good model fit (p = 0.999). After fitting, the logistic regression model allowed us to state that patients with IPAP ≥ 13.5cmH_2_O on the last day of NIPPV support are three times more likely to experience NIPPV failure compared with individuals with IPAP < 13.5 (OR = 3.02, 95%CI = 1.01 - 10.52, p value = 0.040).

## DISCUSSION

The use of noninvasive ventilation after planned extubation is part of clinical practice worldwide.^(1-4,15)^ In a study by Carlucci et al.,^(16)^ the rate of NIPPV in 52 ICUs was 8%. In our hospital, we estimated almost twice that rate (15%). In a cohort study over six years, Harris et al.^(17)^ concluded that the rate of NIPPV use has increased over time. As we observed, the use of NIPPV after extubation is also high. In our study population, NIPPV after extubation accounted for almost half of all NIPPV use. The literature on this issue has presented conflicting conclusions. In summary, randomized clinical trials with a preventive approach had better results, with lower NIPPV failure or reintubation rates, as shown in some studies^(6,10,12,17-20)^ that had reintubation rates from 8 to 11%. On the other hand, Esteban et al.^(14)^ found that NIPPV was not effective for averting ARF after extubation, as they observed a reintubation rate of 48%. Few meta-analyses have focused on NIPPV after extubation. Burns et al.^(7)^ and Zhu et al.^(20)^ concluded that NIPPV had positive effects on mortality and ventilator-associated pneumonia. They also found that there is insufficient evidence to definitively recommend the use of NIPPV to avoid extubation failure^(20)^ and suggested that the benefits of NIPPV on the weaning process need to be elucidated.^(7)^ Glossop et al.^(21)^ concluded that NIPPV reduces the ICU length of stay and instances of pneumonia when used in post-surgical patients and as a weaning method. In addition, they found that it reduces the reintubation rate and length of hospital stay in post-surgical patients, suggesting that NIPPV could be useful for patients who may deteriorate after major surgery. Lin et al.^(22)^ corroborates that NIPPV is not beneficial in those cases, while early NIPPV application after planned extubation decreased the reintubation, ICU mortality and hospital mortality rates.

We estimated that the NIPPV failure rate after extubation was high (34%) and the main cause of NIPPV failure was a new event of ARF. NIPPV failure after extubation did not differ according to NIPPV indication (i.e., ARF initiation).

In randomized clinical trials, we observe that the reintubation rate is lower than in observational studies. Esteban et al.^(14)^ showed a high reintubation rate, but we noticed that the inclusion criteria differed from other studies; specifically, patients were included after ARF initiation. All other randomized clinical trials had a preventive approach and obtained lower reintubation rates. In cohort studies, we observed a reintubation rate of 40% in two studies.^(16,23)^ We noticed that, except for the study by Esteban et al.,^(14)^ randomized clinical trials have presented lower reintubation rates than observational studies. During the period of data collection, the intensive care units included in our study did not have a standardized protocol of weaning or NIPPV use after extubation, and we did not observe any difference between reintubation rates in a group of patients who used NIPPV at an early stage or immediately after extubation.

Because there was not a standardized protocol of weaning, clinical decisions regarding NIPPV parameters, target physiological parameters and reintubation were made by the ICU team. In the hospital where the study was carried out, the ICU team usually follows the recommendations in the literature,^(23)^ such as reintubation in the case of a respiratory rate over 25 breaths per minute, peripheral oxygenation under 90% with high FiO_2_ and pH < 7.25. However, these parameters were not controlled across the units.

Antonelli et al.^(24)^ observed that there are many risk factors for NIPPV failure in ARF and found that a SAPS II score ≥ 35, the presence of acute respiratory distress syndrome and pneumonia were independent factors of failure.

We estimated that levels of IPAP > 13.5cmH_2_O are associated with NIPPV failure. Rana et al.^(25)^ did not find any association between IPAP and EPAP levels and NIPPV outcome. They studied a group of acute lung injury patients in a tertiary care center. However, the IPAP and EPAP levels were not high, with a median IPAP of 12 to 13, and EPAP of 5 to 5.5cmH_2_O. NIPPV parameters were collected from charts during the study course, but we could not identify how that information was managed. Other studies concerning NIPPV after extubation did not evaluate these parameters.^(22)^ Our results showed that failure group patients presented higher levels of NIPPV parameters at the last day of NIPPV use, suggesting that patients with higher NIPPV pressure levels were more likely to fail.

The elevated IPAP levels might indicate that those patients presented unfavorable pulmonary condition, such as poorer respiratory mechanics, muscle inefficiency, higher respiratory work of breathing, higher dead space, or even systemic manifestations that would increase ventilator demand, including metabolic acidosis and shock, although these variables were not controlled in our study. We suggest that IPAP might be a good marker for NIPPV outcome, but our data do not support that IPAP ≥ 13.5cmH_2_O is a cutoff value for NIPPV failure. The heterogeneity of the study population and design are not appropriate to answer this question. On the other hand, these results raise some important questions, such as whether it is possible to identify cutoff values of NIPPV parameters to prevent poor NIPPV outcomes, such as late reintubation.

We observed that patients who experienced a NIPPV failure after extubation presented poorer ICU outcomes, such as a higher tracheostomy rate, longer ICU length of stay and greater mortality rate. Data from the literature are conflicting on this issue, but the studies that we researched have some interesting features that can explain these findings. The results of Esteban et al.^(14)^ and Su et al.^(10)^ are similar. The authors did not find any difference in outcomes, but there was a high NIPPV failure rate (48%) in the study by Esteban et al.^(14)^ and a low extubation failure rate in the study by Su et al.,^(10)^ which was 13% in the control group and 14.9% in NIPPV group. In both studies, we do not observe any advantages of NIPPV, and, obviously, there was no impact on clinical outcomes. On the other hand, the studies that estimated NIPPV efficacy showed improved ICU outcomes. Girault et al.^(6)^ showed that NIPPV reduced the duration of weaning; Ferrer et al.^(9)^ estimated that NIPPV improved the 90-day survival and reduced reintubation rates, and Trevisan et al.^(11)^ found that the use of NIPPV when weaning patients with spontaneous breathing trial failures reduced the pneumonia rate and the need for a tracheostomy.

## CONCLUSIONS

This study was performed at a single university hospital in Brazil, and we believe that our results may not be generalizable. Our results indicate that patients with inspiratory positive airway pressure ≥ 13.5cmH_2_O on the last day of noninvasive positive pressure ventilation support are three times more likely to experience noninvasive positive pressure ventilation failure, and that some points should be considered for future research, such as the identification of a reliable cutoff to better indicate noninvasive positive pressure ventilation discontinuation, based on noninvasive positive pressure ventilation parameters and the patient’s severity, to avoid delayed reintubation and the poor outcomes associated with this procedure.
